# An adult case of atypical familial Mediterranean fever (pyrin‐associated autoinflammatory disease) similar to adult‐onset Still’s disease

**DOI:** 10.1002/ccr3.2102

**Published:** 2019-03-12

**Authors:** Hayato Tsuruma, Hiroe Sato, Eriko Hasegawa, Yukiko Nozawa, Takeshi Nakatsue, Yoko Wada, Takeshi Kuroda, Yoshiki Suzuki, Masaaki Nakano, Ichiei Narita

**Affiliations:** ^1^ General Clinical Training Center Niigata University Medical and Dental Hospital Niigata Japan; ^2^ Division of Clinical Nephrology and Rheumatology Niigata University Graduate School of Medical and Dental Sciences Niigata Japan; ^3^ Health Administration Center Niigata University Niigata Japan; ^4^ School of Health Sciences, Medical Laboratory Science, Faculty of Medicine Niigata University Niigata Japan

**Keywords:** adult‐onset Still’s disease, elevated liver enzymes, Familial Mediterranean fever, pyrin‐associated autoinflammatory diseases, skin rash

## Abstract

We present a 55‐year‐old woman with periodic fever and symptoms similar to adult‐onset Still's disease (AOSD). She had a heterogeneous mutation of the *MEFV* gene and colchicine was effective. Atypical familial Mediterranean fever (pyrin‐associated autoinflammatory disease) should be considered in patients with periodic fever accompanied by symptoms similar to AOSD.

## 1 INTRODUCTION

Familial Mediterranean fever (FMF) is a hereditary autoinflammatory disease characterized by periodic febrile attacks.^1^ Typical cases present with a periodic fever lasting 12‐72 hours associated with serositis, monoarthritis (hip, knee, or ankle), and erysipelas‐like erythema. These symptoms remit spontaneously without specific treatment and colchicine therapy is usually effective for controlling the febrile attacks. By contrast, atypical FMF has shorter or longer febrile periods than in typical FMF, and no serositis or arthritis, and is often difficult to diagnose. Recently, FMF and atypical FMF have been proposed to be under the “roof” of pyrin‐associated autoinflammatory diseases (PAAD).^2^

Clinically, adult‐onset Still's disease (AOSD) resembles FMF because both diseases have spiking fevers, arthritis, and skin rashes. However, the typical skin manifestations are different: a salmon‐pink rash in AOSD and erysipelas‐like erythema in FMF.

Here, we report an adult case of atypical FMF (PAAD) associated with a heterogeneous mutation in exon 3 (P369S/R408Q) of the *MEFV* gene that was similar to AOSD accompanied by a spiking fever, arthritis, elevated liver enzymes, pancytopenia, lymphadenopathy, and various skin rashes.

## 2 CASE REPORT

**Figure 1:**
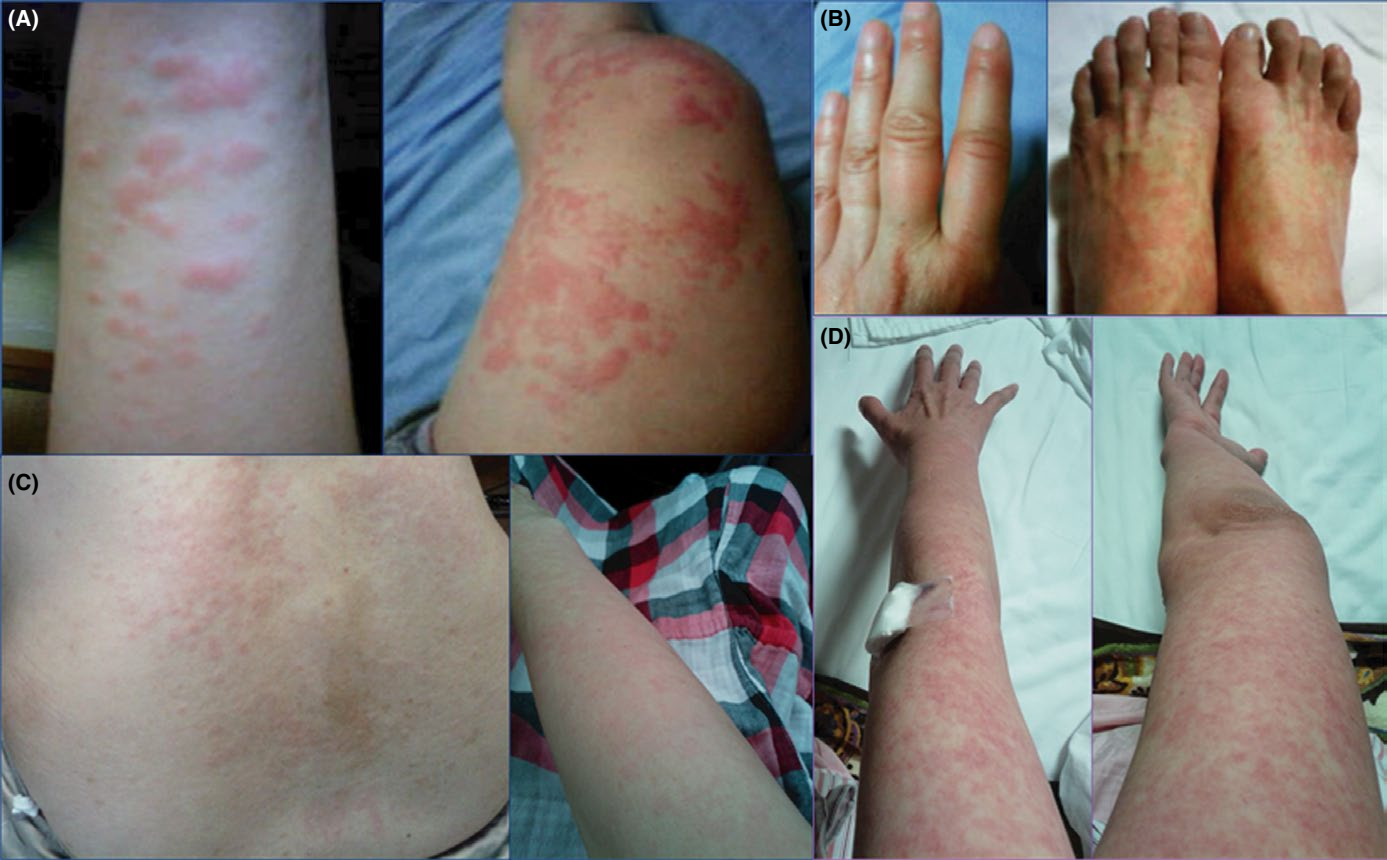
The various rashes seen in our patient: A, urticaria of the arms and legs; B, erythema and swelling of the fingers; C, pink rash on the back and salmon‐pink rash on an arm before admission; and D, erythema multiform of the upper limbs on the fourth day of fever

A 55‐year‐old Japanese woman was admitted to our hospital with a periodic fever and skin rash (Figure 1). She had experienced repeated fevers over 38°C, lasting for 7‐10 days, every 14‐20 days for 2 months. These had occurred several times a year since she was 50 years old. Arthritis accompanied the febrile attacks. Transient signs of a liver injury were detected when she was 50 years old. Cervical and mediastinal lymphadenopathy was detected when she was 51 years old and both improved spontaneously. Figure 1 shows her skin rash, which was pruritic. At times, she developed various rashes, including urticaria of the arms and legs (Figure 1A), erythema and swelling of the fingers (Figure 1B), and a pink rash on her back and a salmon‐pink rash on one arm that appeared and disappeared just before admission (Figure 1C). A skin biopsy was performed, but the findings were nonspecific. She had no family history of periodic fever or autoimmune disease. She was suspected many food allergies, including eggs, flour, milk products, and potatoes, because of her repeated fevers and skin rash. She had been treated with antipyretics and anti‐allergy drugs, but not with immunosuppressive therapy.

On admission, she complained of a productive cough for several days, but her temperature was normal and the skin rash had almost disappeared. Her blood cell counts were normal. The aspartate aminotransferase (AST; 79 IU/L), alanine aminotransferase (ALT; 51 IU/L), and lactate dehydrogenase (LDH; 471 IU/L) levels were slightly elevated, while the alkaline phosphatase (ALP; 152 IU/L) and γ‐glutamyl transpeptidase (γ‐GTP; 24 IU/L) levels were normal. The C‐reactive protein (CRP) level was 2.86 mg/dL and the ferritin level was markedly elevated (4731 ng/mL). Screening tests for hepatitis B and C were negative. Serological markers of Epstein‐Barr virus indicated a postinfectious state. Antinuclear antibody and rheumatoid factor were negative, while anti‐SS‐A antibody was positive. Since Schirmer's test was positive, Sjögren's syndrome was diagnosed.

Computed tomography (CT) showed light patchy shadows in both lungs. Bronchopneumonia was diagnosed and treated with intravenous ceftriaxone (CTRX), and the patchy shadows and productive cough improved. Sixty seven Gallium scintigraphy showed no specific findings.

**Figure 2:**
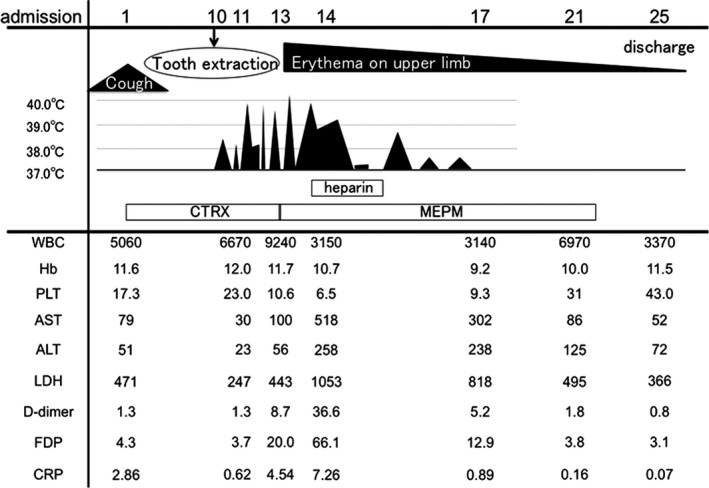
Clinical course of our patient. ALT, alanine aminotransferase (IU/L); AST, aspartate aminotransferase (IU/L); CRP, C‐reactive protein (mg/dL); CTRX, intravenous ceftriaxone; FDP, fibrin degradation products (µg/mL); Hb, hemoglobin; LDH, lactate dehydrogenase (IU/L); MEPM, intravenous meropenem; PLT, platelet count (×10^4^/mm^3^); WBC, white blood cell count (/mm^3^)

To screen for oral infection, she was referred to a dentist, and no infection was evident. However, her third molar was extracted prophylactically 10 days after admission. About 8 hours after the tooth extraction, she developed a fever over 38°C and a spiking fever of 38‐39.5°C that lasted for 6 days (Figure 2). Her CRP level increased to 7.26 mg/dL, with pancytopenia and markedly elevated liver enzymes. D‐dimer and fibrin degradation product levels were also elevated up to 36.6 and 66.1 µg/mL, respectively. Since sepsis complicated with disseminated intravascular coagulation was considered, the CTRX was switched to meropenem (MEPM) and intravenous heparin was started. Blood cultures were negative and echocardiography did not indicate endocarditis. On the fourth day of her fever, erythema multiform appeared on her upper limbs (Figure 1D). Since skin rashes, arthralgia lasting more than 2 weeks, lymphadenopathy and elevated liver enzymes were accompanied by high fever, AOSD with macrophage‐activation syndrome was considered.^3^ Drug fever and antibiotic‐related rash due to CTRX were also considered, but the symptoms did not improve immediately after switching to MEPM. On the eighth day after the fever started, her temperature normalized, apparently spontaneously rather than because of any treatment. The rash and laboratory markers also improved gradually. Variant FMF was suspected because of the periodic fever lasting 7‐10 days without serositis, and colchicine (0.5 mg/d) was started. Febrile attacks or rashes which had occurred several times a year before taking colchicine did not recur in the following 3 years. Genetic studies revealed a heterogeneous mutation in exon 3 (P369S/R408Q) of the *MEFV* gene. She fulfilled Tel‐Hashomer criteria with two minor criteria^1^ as follows; incomplete attacks involving joint and favorable response to colchicine. Atypical FMF (PAAD) was diagnosed.

## 3 DISCUSSION

Familial Mediterranean fever is an autoinflammatory disease. However, the diagnosis of FMF is sometimes difficult in patients with atypical symptoms and features. Our patient had periodic fevers lasting 7‐10 days that were associated with skin rashes and arthralgia for more than 5 years, since she was 50 years old. A tooth extraction triggered a severe febrile attack with a skin rash, elevated liver enzymes, and pancytopenia; this resembled AOSD with macrophage‐activation syndrome. Colchicine dramatically suppressed the febrile attacks and other symptoms.

Recently, PAAD was proposed by a Delphi study.^2^ It includes all diseases associated with pyrin defects or *MEFV* mutations, such as periodic fever with autoinflammation and neutrophilic dermatosis, FMF, chronic nonbacterial osteomyelitis, livedoid ulcerative dermatitis, and novel autoinflammatory diseases with MEFV alterations yet to be determined. Since our patient was Japanese and the mutation was in exon 3 rather than exon 10, PAAD might be a more appropriate diagnosis than atypical FMF.

Adult‐onset Still's disease is a systemic inflammatory disease associated with a spiking fever, skin rash, arthralgia, and elevated ferritin levels. Since autoantibodies are usually negative and it is associated with a severe inflammatory state, AOSD is thought to be an autoinflammatory disease. IL‐18 is a pro‐inflammatory cytokine of the IL‐1 family that is thought to play a key role in the pathogenesis of AOSD.^4^, ^5^ Higher IL‐18 levels are also reported in attack‐phase FMF patients than in healthy subjects, and in patients with an exon 10 *MEFV* mutation relative to those without.^6^

Several studies have investigated the association between*MEFV* gene mutations and AOSD or systemic‐onset juvenile idiopathic arthritis.^7^, ^8^, ^9^ *MEFV* gene mutations were observed more frequently in 20 Turkish AOSD patients (15%) than in healthy controls, but the difference was not significant.^7^ Significantly higher frequencies of exon 10 *MEFV* mutations have been reported in patients with AOSD than in healthy controls.^8^ However, the carriage rate was low (6.1% vs 0%) and the frequencies of the MEFV variants did not differ (63.3% vs 58.1%).^8^ The frequency of a heterogeneous mutation in exon 3 (P369S/R408Q) of the MEFV gene which was observed in our patient was not different between patients with FMF (1.9%‐9.5%) and non‐FMF (1.7%‐7.2%) in Japan^10^ and was reported 6.1% in patients with AOSD.^8^ Recently, colchicine was reported to improve AOSD without any typical mutation of the *MEFV* gene to FMF.^11^ A 24‐years Japanese female with refractory AOSD treated with immunosuppressive agents and biologics was dramatically controlled by adding colchicine.^11^ Thus, the diseases of FMF and AOSD could overlap.

The typical skin manifestation of FMF is an erysipelas‐like eruption on the calves.^1^ Various cutaneous manifestations have also been reported, including urticaria, diffuse erythema on the palms and soles, and subcutaneous nodules.^12^, ^13^, ^14^ Maculopapular eruptions with erythema on the lower extremities and trunk were reported in a patient with atypical FMF associated with a heterozygous E148Q‐P369S mutation of *MEFV*.^15^ In our patient, urticaria, erythema on the fingers, a salmon‐pink rash, and erythema multiforme of the upper limb were observed during different febrile attacks. A salmon‐pink rash is typical in AOSD. Urticaria and pruritic papules or plaques have also been reported as atypical skin manifestations of AOSD,^16^ in 10% of patients.^17^ Therefore, the skin manifestations of FMF and AOSD are similar.

The liver enzymes levels were acutely elevated in our patient following a fever and resolved completely with colchicine. Liver involvement has been reported in 18.9% of children with FMF and all of them were treated effectively with colchicine.^18^ An adult case of acute hepatitis with FMF was also reported, and the histological diagnosis was nonspecific reactive hepatitis with liver cell necrosis and interlobular inflammatory cell invasion.^19^ Liver abnormalities are common in patients with AOSD, occurring in 65% of cases.^4^ The histological findings are similar to those of nonspecific reactive hepatitis.^20^ Inflammatory cytokines, such as IL‐1, IL‐18, TNF, and IL‐6, are thought to play an important role in the liver enzyme elevation seen in both FMF and AOSD.^18^, ^21^

Macrophage activating syndrome and disseminated intravascular coagulation are sometimes associated with AOSD. The frequencies of macrophage activating syndrome and disseminated intravascular coagulation were not different between AOSD patients with or without *MEFV* variants.^8^ But those with FMF have not been reported. One case report was found about an 11‐year‐old girl with tumor necrosis factor receptor 1‐associated periodic syndrome (TRAPS) accompanied with macrophage‐activation syndrome.^22^ Transient laboratory changes indicating macrophage activating syndrome and disseminated intravascular coagulation were seen in our patient, but those improved spontaneously. Such clinical course was different from that of typical AOSD.

Sjögren's syndrome was diagnosed in our patient because anti‐SS‐A antibody and Schirmer's test were positive. There has been a case report of women complicated with FMF (M6941I) and Sjögren's syndrome.^23^ In that case, Sjögren's syndrome was diagnosed because Saxon test, ophthalmologic examination, Schirmer's test and sialography were positive but anti‐SS‐A and anti‐SS‐B antibodies were negative. Periodic fever has also been reported as a manifestation of primary Sjögren's syndrome.^24^ Their patient did not have putative FMF mutations, but the authors did not state which MEFV genotype had been examined. Furthermore, they did not treat the patient with colchicine, so FMF could not be excluded completely. The relationship between FMF and Sjögren's syndrome needs to be investigated in further studies.

Ultimately, this patient met two of the minor criteria of Tel‐Hashomer^1^: incomplete attacks involving joints and a favorable response to colchicine. She also met the AOSD criteria.^3^ As we mentioned above, the frequency of mutations of the *MEFV* gene did not always differ between FMF and non‐FMF patients, and colchicine can be effective in AOSD. Distinguishing between PAAD and AOSD is difficult in some situations, since patients with AOSD were not included in the control group used for establishing the Tel‐Hashomer criteria^1^ and FMF was not recognized when the diagnostic criteria for AOSD were established.^3^ Further study is needed to resolve the pathogenesis of these diseases and determine the optimal for both diseases.

Major limitation of this report was that cholchicine had not been discontinued and we could not completely conclude colchicine absolutely regulate periodic fevers. We did not perform further genetic screening of an autoinflammatory gene panel.

In conclusion, we reported an adult case of atypical FMF (PAAD) that was similar to AOSD and accompanied by elevated liver enzymes, pancytopenia, lymphadenopathy, and various skin rashes; colchicine therapy was remarkably effective. Atypical FMF (PAAD) should be included in the differential diagnosis of patients with periodic fever accompanied by symptoms similar to those of AOSD.

## CONFLICT OF INTEREST

None declared.

## AUTHOR CONTRIBUTIONS

HT, HS EH, YN, TN and YW: helped to collect, analyze, and interpret the data. HT and HS: wrote the initial draft of the manuscript. TK, YS, MN, and IN: assisted in the preparation of the manuscript. All of the authors have critically reviewed the manuscript. All authors approved the final version of the manuscript.
